# The Foreign Journals: Chemistry

**Published:** 1841-04

**Authors:** 


### CHEMISTRY.

*On a peculiar Substance occurring on the Human Teeth.* By F. BUEHLMANN, of Bern.

THOSE bodies, which occur amongst the *tartar* of the teeth, mixed with earthy matter and epithelium cells, were described by Leeuwenhoeck. (Op. omn. i, t. ii, p. 40.) They are filiform, and are found in three different conditions. 1st, beautiful delicate fibres, which seem to sprout from a mass of granular yellowish substance, as the stems of plants do from bulbs, and which are usually collected in tufts; 2d, the same fibres broken and scattered among the epithelium and mucus; and 3d, masses of fibres like those first mentioned, mixed up with the yellowish granular matter.

The first of these forms is the most beautiful, and perhaps the original one. At first the fibres look very like seminal animalcules, as they are sometimes collected in tufts, or like some kinds of fungi. They are about 0·00006 of a Paris inch broad, and their length varies considerably, from about one tenth to one half a line, judging by the author's plates of them. At their free extremities they are gradually acuminated: they are smooth, yellowish white, somewhat transparent, beautifully arched or wavy, and elastic. They break straight across their length, and appear homogeneous.

These fibres occur only on the teeth; they are most numerous where the tooth adjoins the mucous membrane, and especially where a piece of membrane dips into a gap between two teeth; and they are most abundant about teeth that are covered with a tartarous deposit, though not absent on the cleanest. Neither the strongest nitric, sulphuric, nor hydrochloric acid, nor caustic potash produces any change in their appearance beyond that of making them rather more transparent: combustion chars the substance around them, but leaves them quite unaltered.

*Müller's Arhiv. Heft* iv. 1840, p. 442.

